# Automation in the gamete laboratory: have we reached a turning point?

**DOI:** 10.1016/j.xfre.2026.04.007

**Published:** 2026-04-25

**Authors:** Richard J. Paulson

**Affiliations:** USC-HRC Fertility, Division of Reproductive Endocrinology and Infertility, Department of Obstetrics and Gynecology, Keck School of Medicine of the University of Southern California, Los Angeles, California

Everyone knows that the real miracles of in vitro fertilization (IVF) happen in the IVF laboratory. The sight of human eggs, sperm, and embryos in the laboratory invoke, at least in some of us, a sense of incredulity, fascination, even awe. The process of reproduction, normally hidden from view and cloaked in secrecy, suddenly appears. The laboratory environment gives us the opportunity to take a very direct role in the reproductive process. On the other hand, that same environment also makes it abundantly clear how sensitive–indeed, even fragile–the gametes and embryos are. The slightest oversight can make the difference between continued development and demise.

My own fascination with the IVF laboratory began during my fellowship at the University of Southern California, when I was assigned to the practice of Richard Marrs, who had accomplished, in 1982, the second live birth after IVF in the United States. Following him around, trying desperately to learn this completely new field, it was obvious that the most intriguing part of the IVF process lay in the laboratory. At that time, at least in his practice, IVF doctors were expected to perform every part of the newly discovered IVF process themselves. We started in the laboratory, washing sperm, preparing dishes, checking carbon dioxide levels in the incubators. We rushed to the operating room and, with an eye pressed to the laparoscope (cameras were not yet used) performed the difficult laparoscopic procedures that were necessary to access the ovaries and collect the follicular fluid. We carried the fluid-filled test tubes back to the laboratory and identified the oocytes in the follicular aspirates. Richard was a meticulous surgeon, and his dexterity seemed to translate seamlessly to laboratory procedures. This was certainly not the case for me; but I was intrigued, motivated and made a concentrated effort to master as many of the new technical skills as possible.

Every day began in the laboratory. The appreciation for the precision and attention to detail was an integral part of the practice. It was akin to building cars by hand, rather than on an assembly line: slow, meticulous, frequently gratifying, but clearly inefficient. One good part was that we were intimately aware of the progress of every embryo, and I valued being able to tell patients, “Yes, I looked at your embryos today and they’re doing great!”

Gradually, however, work in the IVF laboratory became more complex. Embryo cryopreservation, assisted hatching, intracytoplasmic sperm injection (ICSI), and embryo biopsy, all technically demanding procedures, represent a form of microsurgery, or “single-cell surgery.” Laboratory responsibilities were increasingly assumed by specially trained embryologists, who are, in effect, high volume microsurgeons. Their focus and repetition, like that of master watchmakers, allow them to achieve outcomes far superior to those of individuals who perform these tasks only occasionally. As the workload increased, the IVF laboratory evolved toward a more efficient assembly-line model, and it is here that automation logically entered the picture.

The fully automated IVF laboratory no longer is merely theoretical (1, 2). Powered by artificial intelligence, the precise, technically demanding, yet repetitive tasks of embryologists may be programmed, refined, and improved (3). Automation allows specific procedures to be tested, evaluated, and optimized, while ensuring consistency and minimizing drift. Although automation has not yet surpassed the dexterity of the high-volume microsurgeons of the IVF laboratory, it soon may do so. I have long anticipated the IVF laboratory of the future, and it now appears that we are getting close. Two publications from February of this year suggest that we have, at the very least, reached a turning point.

The first report described microfluidic isolation of oocytes from follicular fluid aspirates (4). Successfully isolating viable, mature oocytes without harm would itself represent a remarkable achievement. However, the investigators went beyond proof of concept. Their system was able to surpass human performance, identifying additional oocytes in samples that already had been screened by experienced embryologists. The findings were striking: among 582 patient samples, 54.3% contained additional oocytes, yielding a total of 583 previously undetected oocytes, 41.2% of which were mature. These oocytes, missed by human hands and eyes, were viable and capable of resulting in live births.

The second study (5) reported the first live births resulting from a sequence of automated “day 0” procedures: oocyte identification, cumulus denudation, sperm preparation, and intracytoplasmic sperm injection. Automation of each of these procedures had been reported previously, but this was the first demonstration that they could be used in sequence leading to live births. This is a critical advance, as a fully functional laboratory must link individual processes into a cohesive system. If this integration can be achieved, it is not difficult to envision additional procedures being incorporated into the sequence, including trophectoderm biopsy, vitrification, and thawing.

Perhaps the transition to automation once again will allow clinicians like me to feel like they are participating in the thrill of reproduction in vitro. We will be able to visit the IVF laboratory without fear of disrupting delicate work or causing a catastrophe by bumping into an embryologist carrying a stack of laboratory dishes—my personal nightmare. We will be able to monitor, perhaps even remotely, the progress of the gametes and embryos in the laboratory. We will be able to observe the entire process, from oocyte identification to blastocyst formation.

And perhaps, in this new era, we will once again be able to tell our patients, “Yes, I looked at your embryos today and they are doing great!”
